# A systematic review of treatment for patients with burning mouth syndrome

**DOI:** 10.1177/03331024211036152

**Published:** 2021-08-18

**Authors:** Huann Lan Tan, Jared G Smith, Jan Hoffmann, Tara Renton

**Affiliations:** 1 Faculty of Dentistry, Oral & Craniofacial Science, King’s College London, London, UK; 2 Faculty of Dentistry, The National University of Malaysia, Kuala Lumpur, Malaysia; 3 Population Health Research Institute, St George’s, University of London, London, UK; 4 Wolfson Centre for Age-Related Diseases, Institute of Psychiatry, Psychology and Neuroscience, King’s College London, London, UK; 5 NIHR-Wellcome Trust King’s Clinical Research Facility/SLaM Biomedical Research Centre, King’s College Hospital, London, UK

**Keywords:** Burning mouth syndrome, glossodynia, treatment, systematic review

## Abstract

**Background:**

Burning mouth syndrome is a chronic idiopathic intractable intraoral dysaesthesia that remains a challenge to clinicians due to its poorly understood pathogenesis and inconsistent response to various treatments.

**Aim:**

This review aimed to study the short- (≤3 months) and long-term (>3 months) effectiveness and sustainable benefit of different burning mouth syndrome treatment strategies and the associated side effects.

**Materials and methods:**

Randomised controlled trials of burning mouth syndrome treatment compared with placebo or other interventions with a minimum follow up of 2 months were searched from the PubMed, Embase and Cochrane database (published to July 2020).

**Results:**

Twenty-two studies were selected based on the inclusion and exclusion criteria and analysed. Nine categories of burning mouth syndrome treatment were identified: Anticonvulsant and antidepressant agents, phytomedicine and alpha lipoic acid supplements, low-level laser therapy, saliva substitute, transcranial magnetic stimulation, and cognitive behaviour therapy. Cognitive behaviour therapy, topical capsaicin and clonazepam, and laser therapy demonstrated favourable outcome in both short- and long-term assessment. Phytomedicines reported a short-term benefit in pain score reduction. The pooled effect of alpha lipoic acid (ALA) pain score improvement was low, but its positive effects increased in long term assessment.

**Conclusion:**

A more significant volume in terms of sample size, multi-centres, and multi-arm comparison of therapeutic agents with placebo and longitudinal follow-up studies is recommended to establish a standardised burning mouth syndrome treatment protocol. Further studies are required to assess the analgesic benefits of topical clonazepam and capsaicin, alternative medicines with neurodegenerative prevention capability and psychology support in treating burning mouth syndrome and reducing systemic adverse drug reactions.

**Registration** International Prospective Register of Systematic Reviews (PROSPERO):

Protocol ID - CRD42020160892.

## Introduction

Burning mouth syndrome (BMS) is defined as idiopathic orofacial pain with intraoral burning or dysaesthesia recurring daily for more than 2 hours per day and more than 3 months, without any identifiable causative lesions, with and without somatosensory changes in International Classification of Orofacial Pain, 2020 (1). BMS prevalence ranges from 0.1% to 3.9% and is primarily present in postmenopausal women aged between 50 and 70 (2,3). BMS commonly manifests as burning, prickling, tingling, itching or numbness affecting the tongue, lip, palate, gums and other oral mucosae (4). The pain intensity increases throughout the day and peaks in the late evening (5). Patients often complain of dysgeusia, xerostomia, altered sensation in the oral mucosa, and psychological issues such as anxiety and depression. The pathogenesis of BMS has been hypothesised to be associated with psychological disorders (6) and peripheral and central neuropathy (7), but at present it is classified as idiopathic chronic pain (1). Diagnosing and managing patients with BMS remains a challenge to clinicians due to its poorly understood pathogenesis and inconsistent and limited response to various treatments. Besides, it has an exceptionally low spontaneous remission prevalence of 3–4% after 5–6 years of diagnosis (8). There are no global guidelines on BMS treatment, and published review articles included clinical studies with limited follow up periods (<2 months) (9–11). Based on the current universal ICOP criteria, the diversity of BMS patients’ underlying pain mechanism, and the difference in evidence on short- and long- term benefit of treatment in BMS (11), we sought to conduct a systematic review on different therapeutic strategies for patients presenting with BMS, with the question “which range of treatments have effective short (≤3 months) and long-term (>3 months) outcomes in improving the pain symptoms in BMS patients?”. Parallel with the aim of providing a personalised treatment for each patient, the sustainability of a treatment efficacy and patients’ compliance and response towards the therapy and its side effects should be considered.

## Methodology

### Search strategy

The study was carried out following the PRISMA guidelines (12). An electronic search on PubMed Medline (1946 to 1 July 2020), Embase Ovid (1980 to 1 July 2020), Cochrane Database of Systematic Reviews (1 July 2020) and Cochrane Central Register of Controlled Trials (CENTRAL) (1 July 2020) was conducted based on the combination of the following keywords: “burning mouth syndrome or glossalgia or stomatodynia AND treatment or therapy or therapeutic or management”. This review includes all randomised and controlled clinical trials with a placebo published in the English language. The included studies should state that the diagnosis of BMS is based on the absence of local and systemic pathological contributing factors and have a minimum follow up of treatment of 2 months. This systematic review was registered in PROSPERO (Protocol ID: CRD42020160892). We also performed a manual search on all included clinical trials in published systematic review articles for any potentially relevant studies.

### Study selection

The search results were screened based on the relevant title and abstract by two independent authors. Where information from the abstract was inadequate to allow a decision, a full report was obtained. The full text was obtained for articles fulfilling the inclusion criteria. Any disagreements were resolved by discussion between the authors, and the review authors were not blinded to articles’ authorship. Studies meeting the inclusion criteria underwent data extraction and were evaluated for study risk of bias. The following data were obtained and recorded in a standardised proforma sheet on author and year of publication; study design or methodology; sample size and participant inclusion and/or exclusion criteria; types of intervention and follow-up time; the outcome and/or adverse effect from the intervention; statistical methods employed (Table 1).

**Table 1.** Summary of included studies and quality of evidence.

Author/Year	Intervention	Sample size; Mean age (years) (study/control)	Outcome assessment method	Finding summaryShort term (≤3 months)	Long term (>3 months)	Adverse Effect	Quality of Evidence (Grade)
(20)	Clonazepam (0.5 mg)Dosage: 0.5 mgDurations: 9 weeksRoute: Oral	10/10; 67.5/65.4	• Numerical pain ratings (0–10)• BDI• ZMS• Taste test• Smell test• Salivary flow rate	• NPS: Significant difference between clonazepam (MD: 2.9) and placebo (MD: −1.5), (*p* = 0.011)• Taste and saliva: Clonazepam group show significant increase in taste score (*p* = 0.023) and salivary flow (*p* = 0.033)• No significant difference between clonazepam and placebo in taste (*p* = 0.83) and salivary flow (*p* = 0.060)• Depression and mood. No significant difference		No side effect on psychology.	Moderate
(19)	Clonazepam (0.5 mg)Dosage: 0.5–2.0 mg/dayDuration: 6 monthsRoute: Topical. Dissolved in mouth and spat out after 3 min	33/33; 64.9 /64.9	• VAS		• Significant decrease in VAS for Clonazepam (MD: −4.7)• 23 study group improved more than 50% (*p* < 0.05) and three were totally asymptomatic• Not significant decrease in control group (MD: −3.2)• Reduced in tasted alteration and dryness in clonazepam group	Test group: Five had sleepiness but did not require termination of treatment	Moderate
(39)	Clonazepam (2 mg) Dosage: 2 mg/dayDuration: 4 months Route: Oral Pregabalin (150 mg)Dosage: 150 mg/dayDuration: 4 monthsRoute: Oral ALA (600 mg) Dosage: 600 mg/dayDuration: 4 monthsRoute: Oral	25; 4325;4525;42	• VAS		• Significant reduction in VAS score clonazepam (MD: −4.1, *p* < 0.001)• Significant reduction in pregabalin VAS score (MD: −4.7, *p* < 0.001)• No significant reduction in VAS score ALA (MD: −0.7)	• Four dizziness, two transient diarrhoea, two myalgia• Three increased appetite, one vertigo, one mild nausea, one diarrhoea• Two mild nausea, one myalgia	Very low
(21)	Trazodone (100 mg) Dosage 1: 100 mgDuration 1: 4 daysDaily for 4 days Dosage: 200 mg Duration: 8 weeks Route: Oral	11/17; 61.1/NA	• VAS• MPQ• BDI• Global assessment	• Eight in study group and 13 in placebo reported reduction in pain• Pain intensity was significant decreased in (*p* < 0.01) in both trazodone (MD: −1.4) and placebo group (MD: −1.3)• No significant difference between both groups in treatment effect and treatment by time interaction for pain intensity• No significant differences between the groups MPQ for influence of pain on eating, speaking, sleeping or for the suffering caused by the pain• No significant difference between both group in the patient’s global assessment of improvement or benefits of the treatment• Significant decreased in BDI for both groups in the depression score (*p* < 0.01)		Significant dizziness (*p* < 0.001) and drowsiness (*p* < 0.05) in trazodone group than placebo Test group:• 11 reported dizziness, nine drowsiness, five abdominal pain, three headache, two palpitation, two tremor, three dry mouth and one urinary incontinence	Moderate
(30)	Crocin (15 mg)Dosage: 30 mg/dayDuration: 11 weeks Route: Oral Citalopram (10 mg)Dosage: 10 mg for first week followed by 20 mg dailyDuration: 11 weeks. Route: Oral	26; 52.921; 49.0	• VAS• HAD• DSM IV psychiatric diagnosis	• No significant difference in VAS mean score between crocin and citalopram (*p* = 0.98)• Significant reduced in crocin VAS (MD: −7.8, *p* < 0.001) Average recovery percentage of burning mouth score at the end of treatment for both crocin and citalopram was 87.45%• No significant difference in depression and anxiety between crocin and citalopram (*p* = 0.76) • Average recovery percentage of depression score at the end of treatment for crocin was 30.57% and citalopram was 30.79%• Average recovery percentage of anxiety score at the end of treatment for crocin was 15.44% and citalopram was 15.40%			Low
(22)	ALA (200 mg) Dosage: 600 mg/dayDuration: 2 monthsRoute: Oral Participant showing deterioration of symptoms within 4 months will be given another 1 month of supplement	30/30; 45 /NA	• BMS symptomatology change scale (worsening; unchanged; slight improvement; decided improvement; resolution)	• Statistically significant symptom improvement with ALA (97%) compares with control (40%)• 87% ALA patients showed resolution or a decided improvement in symptom but none in control• None of ALA group has worsening of BMS symptoms but 20% in control	• 73% of ALA remains significantly stable (no changes) but 83% of control group shows significant deterioration	No adverse effect reported	Low
(24)	ALA Dosage: 800 mg/dayDuration: 8 weeksRoute: Oral	23/16; 67/59.3	• VAS	• No significant difference (*p* = 0.14) between ALA (MD: 2.2) or control (MD: 3.8)		Test group: One patient has gastrointestinal upset.	Low
(26)	ALA (200 mg) Dosage: 600 mg/dayDuration: 2 monthsRoute: Oral	29/25; 62.13/ 62.13	• VAS • Symptoms response categories• (improvement; no change; worse)	• 64% in ALA and 27.6% in control group reported improvement of symptoms• No ALA patients and five control patients reported worsening of symptoms• Statistically significant differences between both groups (*p* = 0.009)			Low
(23)	• ALA (400 mg) and vitamin (C, PP, E, B6, 2, 1, 12, and folic acid)Dosage: 800 mg/dayDuration: 8 weeksRoute: Oral• ALA (400 mg)Dosage: 800 mg/day Duration: 8 weeks• Route: Oral	18/20; 67.3/NA 14/20; 67.3/NA	• VAS• Weighted MPQ	• Significant reduction in pain intensity (VAS) for studies ALA and vitamin (MD: −0.95, *p* = 0.047) and ALA (MD: −1.79, *p* = 0.045)• ALA + vitamin: One of 18 improved and 17 of 18 no change or worse• ALA: Four of 14 improved and 10 of 14 no change or worse• No significant difference between both study groups and control group• Improvement in MPQ score with high placebo effect observed• No significant difference between all three groups in MPQ	• Significant reduction in pain intensity (VAS) for studies ALA and vitamin (MD −1.78, *p* = 0.047) and ALA (MD: −2.00, *p* = 0.045)• ALA + vitamin:• Six of 18 improved and 12 of 18 no change or worse• ALA:• Four of 18 improved and 14 of 18 no change or worse• No significant difference between both study groups and control group• No significant difference between three groups in MPQ	No adverse effect reported	Moderate
(25)	• ALA Dosage: 600 mg/dayDuration: 60 days Route: Oral • GABADosage: 300 mg/dayDuration: 60 daysRoute: Oral • ALA and GABADosage: 600 ALA and 300 GABA/dayDuration: 60 daysRoute: Oral	ALA:20GABA: 20ALA and GABA: 20Control: 60;57.5/NA	• Numerical category of burning scale:• Category 1: negative changes (deterioration)• Category 2: no changes• Category 3: with positive changes (improvements)• Category 4: with total recovery	• ALA: • Negative: 0%; No change: 45%; positive and total recovered: 55%• ALA 7× higher than control group• GABA:• Negative: 0%; no change: 50%; positive and total recovered: 50%• GABA 5.7× higher than control group• ALA + GABA: Negative: 0%; no change: 30%; positive and total recovered: 70%• ALA + GABA 13.2× higher than control group• Significant level of positive burning changes between group (*p* < 0.001)		Adverse effects appeared very mild.	Moderate
(33)	• ALA (400 mg)Dosage: 800 mg/dayDuration: 8 weeksRoute: Oral• Capsaicin (250 mg chilli powder in 50 ml)Dosage: 750 mg/150 ml/dayDuration: 8 weeksRoute: Topical – oral rinse• Lysozyme lactoperoxidase (Biotene) Fiive times per day Duration: 8 weeksRoute: Topical – oral rinse	*Size* Short term:ALA: 14Capsaicin: 14Biotene: 14Control: 14Long term: ALA: 9 Capsaicin:9Biotene: 9*Age* ALA: 64Capsaicin: 62Control:62	• VAS	• Significant improvement in VAS (*p* < 0.001) ALA: 57% improved (MD: −2.1). Capsaicin: 76% improved (MD: −3.2) Biotene: 57% remain unchanged (MD: −1.7)• No statistically difference in VAS between groups ALA, capsaicin and Biotene• No significant difference VAS improvement in control group• All study groups, ALA, capsaicin and biotene, were statistically superior to control groups	• Only capsaicin group shows significant reduction in VAS score (MD: −2.9) with 67% improved• ALA (MD: −1.8) and Biotene (MD: −1.8) failed to show statistically significant of VAS score improvement with 55% remain unchanged for Biotene and 55% improved with ALA• No difference in trend of VAS in control group	No adverse effects were reported for capsaicin	Low
(31)	Ultramicronised Palmitoylethanolamide (umPEA) (600 mg) Dosage: 1200 mgDuration: 60 daysRoute: Sublingual	13/16NA	• NRS (scale 0 to 10)	• Significant decreased of spontaneous burning intensity between umPEA group (MD −3.8) and control group (MD: −1.3) (*p* = 0.001)	No statistically significant difference between umPEA (MD: −2.4) and control group (MD: −1.4).	No side effect observed	Low
(29)	Herbal catuama (310 mg) Dosage: 620 mg/dayDuration: 8 weeks Route: Oral	30/30; 63.6/61.5	• VNS (0–10)• Faces scale (FS) (Scale 0 to 5) – lesser is better	VNS: • At 8 weeks reduction in symptoms of test group was 52.4% and control group 24.2%• At 12 weeks after treatment onset, 51.3% reduction of symptom, and control reduction 18.8%• Significant difference between test group and control group at 8 weeks (*p* = 0.003) and 12 weeks (*p* = 0.001)FS• Significant difference between test group and control group at 8 weeks (*p* < 0.001) and 12 weeks (*p* = 0.001)		Test group: One patient with somnolence and weight gain, • one insomnia,• three exacerbation of symptoms in first week of treatment	Moderate
(28)	Hypericum perforatum (300 mg) Dosage: 900 mg/dayDuration: 12 weeksRoute: Oral	19/20; 65.9/63.9	• VAS• Number of oral mucosa sites • Quality of health questionnaires (QOH).	• No significant difference between study (MD: −1.8) and control group (MD: −1.1) in VAS (*p* = 0.222)• Significant reduction in number of burning sites in study group• Both groups showed a better QOH and ability to cope with their symptoms at the end of trial		Test group: One had severe headache in the fifth week of therapy	Moderate
(27)	Lycopene-enriched extra virgin oil (300 ppm) Dosage: 900 ppm/dayDuration: 12 weeks. Routes: Topical spray and swallowed	26/24; 61.7/64.9	• VAS* (grade 1 to 10)• SF-36 • OHIP-14• HAD • Patient Rated Benefit and Satisfaction	• Significant reduction in VAS score in both pain (MD: −3.0; *p* < 0.001) and burning (MD: −1.0; *p* = 0.003) symptoms• No significant differences between study and control group in VAS, SF-26, OHIP-14, HAD and Patient Rated Benefit and Satisfaction		No adverse effect reportedNo significant changes in participants’ lipid profile during the 12-week study period	Low
(18)	N-acetyl-5-methoxytryptamine. Melatonin (MLT) (3 mg)Dosage: 12 mg /dayDuration: 8 weeksRoute: Oral	16/16; 64.4/64.4	• VAS • Number of sites• Patient global impression of pain changes• Symptom response categories (worse; no change; mild improvement; moderate improvement; strong improvement)• MOS • HRS	• No significant difference between MLT (MD: −0.6) and placebo (MD: −1.1) group in VAS score• Four MLT group and three control group reported improvement in pain changes• Overall, no change in the number of oral sites affected by pain was recorded• Decrease in the sleep scores for both groups but not statistically difference in sleep impairment between MLT and control group• Non-significant difference in Epworth• Sleepiness Scale (ESS) for diurnal sleepiness• Statistically significant decrease in anxiety for melatonin group (*p* < 0.05)		Test group: 40% of patients dropped out because of sideeffects: Four self-reported heavy tremor, sexual disturbances, blurred vision, and severe heavy-headiness; three lack of efficacy or pain improvement; one lost to follow-up.	Moderate
(34)	Low level laser therapy: IR1W: 830 nm wavelength, 100 mW output power, continuous emissions, 3.57 W/cm^2^, 5 J energy per point, 176 J/cm^2^ radiant exposure, application time 50 sec per point. Duration: One session per week for 10 weeks. Total 10 sessions. IR3W: 830 nm wavelength, 100 mW output power, continuous emissions, 3.57 W/cm^2^, 5 J energy per point, 176 J/cm^2^ radiant exposure, application time 50 sec per point. Duration: Three sessions per week for 3 weeks. Total nine sessionsRed laser: 685 nm wavelength. 35 mW output power, continuous emissions, 1.25 W/cm^2^, 2 J energy per point, 72 J/cm^2^ radiant exposure, application time 58 sec per point. Duration: Three sessions per week for 3 weeks. Total 9 sessions	20/19; 63.6/61.520/19;60.5/61.519/19;63.2 /61.5	• VAS (0–100)• VNS (0–10)• OHIP 14	VNS: • Significant difference between IR1W laser (MD: −4.45) and control (MD: −2.53) at 11 weeks, *p* = 0.005VAS: • Significant difference between IR1W laser (MD: −49.2) and control at 11 weeks; *p* = 0.004.VNS: • Significant difference between IR3W laser (MD: −5.1) and control (MD: −2.53) at 11 weeks; *p* < 0.0001VAS: • Significant difference between IR3W laser (MD: −53.0) and control at 11 weeks; *p* < 0.0001VNS:• No significant difference between red laser (MD: −3.74) and control (MD: −2.53) at 11 weeks; *p* = 0.12VAS:• No significant difference between red laser and control at 11 weeks, *p* = 0.13• No significant difference between IR1W, IR3W and red laser group		No adverse effect reported	Low
(36)	Low level laser therapy (LLLT) Dosage: 810 nm wavelength, 12 J/cm^2^ per session in a continuous modeDuration: Twice a week session for 5 weeks consecutively Total 10 sessions	10/10;60.3/67.6	• VAS• SF-36• OHIP14• EES • SCL 90-R• MPQ		VAS:• 90% LLLT and 20% control reported improvement• Pain decreased significantly (*p* = 0.005) in the study group (MD: −2.9) versus control group (MD: 0.5)McGill:• No significant difference between LLLT and control group in PRI, NWC and PPIOHIP: • Non-significant reduction in LLLT (MD: 4.0, *p* = 0.31).• No significant difference between LLLT and control (*p* = 0.27)SF-36: • No significant difference between LLLT and control group in all categoriesEES:• No significant difference in LLLT (MD: −0.1, *p* = 0.83) and between control (*p* = 0.32)SCL-90-R:• Significant difference in interpersonal susceptibility (*p* = 0.02) and decrease in anxiety (*p* = 0.05) in LLLT group	N/A	Low
(32)	Urea 10%3–4 times dailyDuration: 3 monthsRoute: Topical	12/13; 66.3/58.4	• EDOF-HC • Xerostomia questionnaire• QST	• Seven in study group and eight in control group have reduction in pain• No difference between study and control group pain intensity (*p* = 0.88); salivary flow (xerostomia) (*p* = 0.32); gustation (sweet *p* = 0.38, salty *p* = 0.69, sour *p* = 0.69, bitter *p* = 0.69); olfaction (*p* = 0.98); corneal reflex right (*p* = 0.20) and left (*p* > 0.99)• No significant differences in the somatosensory test between group (*p* > 0.05)		N/A	Low
(35)	Transcranial magnetic stimulation (rTMS) Dosage: total of 30,000 pulses Duration: 10 sessionsRoute: Transcranial	12/8; 63.4/64.4	• VAS • BPI• SF- MPQ• PHQ-9• PGIC• CGI-I	VAS:• Significant reduction in pain with rTMS group (MD: −3.1, *p* = 0.002)• 75% reported > 50% decrease in BMS pain intensity• Significant difference between rTMS and sham group (MD: −2.8, *p* = 0.005)BPI:• Significant improvement for rTMS group (MD: −2.1, *p* = 0.003) and not in control groupSFMPQ:• Non-significant difference in affective score and present pain intensity in rTMS (MD: −1.2) and sham (MD: −0.8) groupPHQ-9:• No significant difference in rTMS (MD: −5.6) and sham group (MD: −1.0)PGIC:• Significant difference in rTMS (MD: 3.3, *p* < 0.01) but not in sham group (MD: 1.4)CGI-I:• Significant improvement in rTMS (MD: −2.3, *p* < 0.01) but not in sham group (MD: −0.62)		Seven in rTMS group and five in sham group had headache at the beginning treatment but very mild and tolerated and disappeared in one or two days	Low
(38)	Tongue protectorDosage: 15 min 3 times/dailyDuration: 2 monthsRoute: Oral appliance	25/25; 61.0/61.4	• VAS• HAD• OHIP-49• SF-36	• VAS: Significant difference (*p* < 0.001) between active (MD: −3.6) and control (MD: −1.4) group• HAD: Depression• Non- significant (*p* = 0.205) between active (MD: −1.0) and control groups (MD: −0.04)Anxiety• Non- significant (*p* = 0.69) between active (MD: −0.1) and control groups (MD: −0.2)• OHIP-49: Significant difference (*p* = 0.008) between active (MD: −18.4) and control (MD: −1.9)• SF-36: Significant difference (*p* < 0.05) between active and control group in physical role, bodily pain, general health, emotional role• Tongue protector group has better oral health (OHIP) and quality of life (SF-36)		No adverse effect observed	Very Low
(37)	Cognitive therapy (CT) One hour once a week A total of 12–15 sessions	15/15	• VAS (1–7)	• Significant reduction in pain symptoms (VAS) in CT group (MD: −2.8, *p* < 0.001) than control group	• Significant reduction in CT group pain symptoms (VAS) (MD: −3.6, *p* < 0.001) than control group	N/A	Low

VAS: Visual analogue scale; VNS: visual numerical scale; NRS: Numeric rating scale; MPQ: McGill Pain Questionnaire; BPI: Brief Pain Inventory; EDOF-HC: Orofacial Pain Clinic Questionnaire; SF-36: 36-Short Form Health Survey (SF-36); OHIP 14: Oral Health on Quality of Life; HAD: Hospital Anxiety and Depression Scale; BDI: Beck Depression Inventory; ZMS: Zerssen Mood Scale; PGIC: Patient Global Impression of Change; CGI-I: Clinical Global Impression for global Improvement Scale; EES: Epworth Sleepiness Scale; PHQ-9: Patient Health Questionnaires-9; HRS: Hamilton Rating Scale; SCL-90-R: Symptom Checklist-90-R; MOS: Medical Outcomes Survey of Sleep Scale; QST: Quantitative somatosensory testing; MD: mean difference from base line; ALA: Alpha lipoic acid; GABA: Gabapentin; N/A : Not available.

### Assessment of risk of bias

We used the Cochrane risk of bias assessment tool (13), which is based on seven main domains (Table 2). Each study was categorised based on the overall risk category and classified as low, unclear, or high risk. The quality of all included articles was assessed using the GRADE (14).

**Table 2.** Risk of biased analysis of included studies.

		Random sequence generation	Allocation concealment	Blinding of participants	Blinding of outcome assessors	Incomplete outcome data	Selective reporting	Other bias
Treatment	Author							
Clonazepam Systemic (oral)	Heckmann et al. 2012	+	+	+	?	+	−	−
	Çinar et al. 2018	?	−	−	?	+	+	?
ClonazepamTopical (rinse)	Rodriguez de Rivera-Campillo et al. 2010	+	?	+	+	+	+	−
Pregabalin	Çinar et al. 2018	?	−	−	?	+	+	?
GABA	Lopez-D'alessandro et al. 2011	+	+	?	+	+	−	+
Trazodone	Tammiala-Salonen et al. 1999	+	+	+	+	?	?	?
Citalopram	Pakfetrat et al. 2019	?	−	+	+	+	+	?
Crocin	Pakfetrat Aet al. 2019	?	−	+	+	+	+	?
								
ALA	Femiano et al. 2002	?	−	+	?	+	−	?
	Lopez-Jornet et al. 2009	+	+	+	+	−	−	?
	Palacios-Sanchez et al. 2015	?	?	+	+	−	−	−
	Carbone et al. 2009	+	−	+	+	?	?	?
	Lopez-D'alessandro et al. 2011	+	+	?	+	+	−	+
	Marino et al. 2010	+	−	?	?	+	+	−
	Cinar et al. 2018	?	−	−	?	+	+	?
ALA + Vitamin	Carbone et al. 2009	+	−	+	+	?	?	?
ALA + GABA	Lopez-D'alessandro et al. 2011	+	+	?	+	+	−	+
Capsaicin Topical (Rinse)	Marino et al. 2010	+	−	?	?	+	+	−
Ultramicronised palmitoylethanolamide	Ottaviani et al. 2019	+	−	+	?	?	−	+
Herbal catuama	Spanemberg et al. 2012	+	+	+	+	−	+	?
Hypericum perforatum	Sardella et al. 2008	+	+	+	+	+	?	+
Lycopene-enriched extra virgin oil	Cano-Carrillo et al. 2014	+	+	+	+	?	−	−
Melatonin	Varoni et al. 2018;	+	+	+	+	?	+	?
Low level laser therapy	Spanemberg et al. 2015	?	?	?	+	+	?	?
	de Pedro et al. 2020	?	−	+	?	+	+	?
Urea Topical (Rinse)	da Silva et al. 2014	?	−	+	?	−	−	?
Lysozyme lactoperoxidase Topical (Rinse)	Marino et al. 2010	+	−	?	?	+	+	−
Transcranial magnetic stimulation	Umezaki et al. 2016	+	?	+	−	−	?	?
Tongue protector	Lopez-Jornet et al. 2011	+	?	−	−	+	+	?
Cognitive therapy	Bergdahl et al. 1995	?	−	−	−	+	+	+
								

ALA: alpha lipoic acid; GABA: gabapentin; ‘?’: unclear risk, ‘+’: low risk; ‘– ‘: high risk

### Outcome analysis

We analysed outcome data based on short term (≥2 month to ≤3 months) and long term (>3 months) changes in symptoms. The assessment method used in the included studies should be of equal measure. The standardised mean difference (SMD) in pain score (VAS) of treatment groups and placebo and their relative risk ratio (RR) for BMS pain improvement was recorded from the relevant studies with the 95% confidence interval (CI) where possible. Estimates of effect (and associated CI) were combined and pooled for studies reporting the same treatment.

### Statistical analysis

Mean difference (MD) of the pre- to post-treatment VAS change scores were extracted from studies. For each study with comparisons between treatment and placebo at short term (≤3 months) and/or long term (>3 months), standardised mean differences (SMDs) of the VAS scores were calculated using pre-to-post-intervention change score (means) and post-intervention SDs (rather than change score SDs which were not provided in several studies). Means and/or standard deviations for baseline and post-treatment pain intensity were calculated for two studies based on the length of error bars in graphs and a ruler and two other studies using raw data (provided in papers). Continuous data were pooled using the Hedges g statistic as a formulation for the SMD under the fixed effects model. For categorical (dichotomous) outcomes (e.g. n ≥ vs. n < 50% decrease in VAS pain intensity, or number of patients demonstrating improvement from baseline versus the number showing no change/worsened score), relative risks (RRs) and associated 95% CI were calculated to express the estimate of treatment effect (15). Where zeros caused problems with the computation of the RR or its CIs, 0.5 was added to frequency cells (16,17). Where appropriate, RR data were pooled (under a fixed effect model). Formal meta-analyses were not performed in this review due to the heterogeneity of the included studies’ methods and outcome data such as varying assessment times within short- and long-term testing periods, differences in treatment regime (e.g. timing or dosage of medication administration), different outcome assessments of burning or general pain improvement, and incomplete data (e.g. variance not reported).

## Results

**Figure 1.**
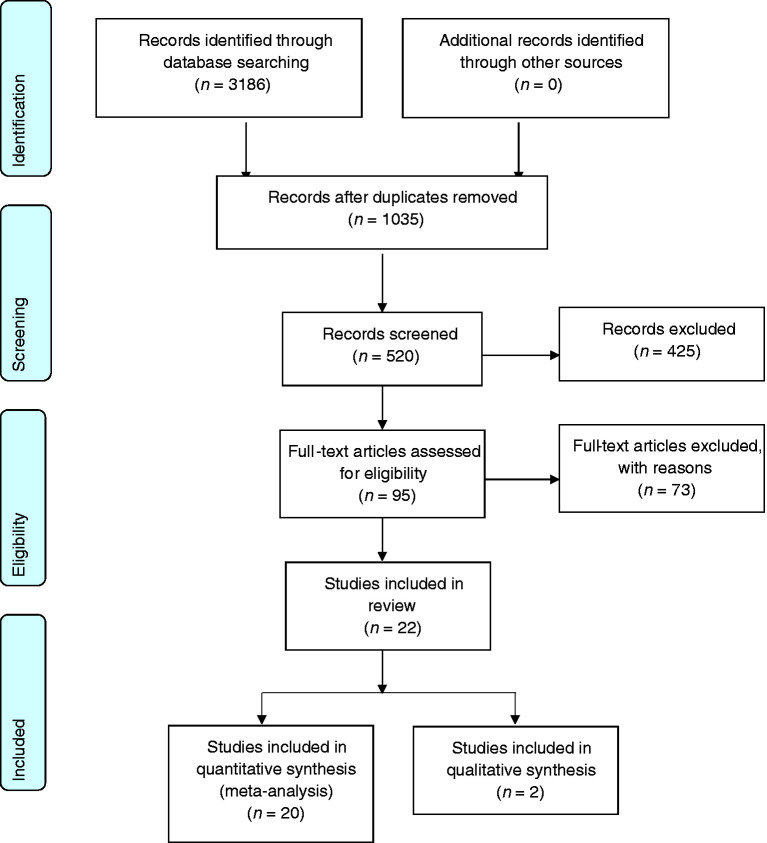
Flow chart on the study selection process (adapted from PRIMA, 2009).

A total of 95 full text published articles were reviewed; 22 were included in this review (Table 1), and 73 were excluded (Table 3). Figure 1 shows the study selection flow process.

**Table 3.** Reasons for studies’ exclusion.

Author	Reason for exclusion
1. Okayasu et al. 2020.	Non randomisation. No control. Follow up at 4 weeks
2. Paudel et al. 2020	Non randomisation. Retrospective study. No control
3. Diep et al. 2019	Non randomisation. Case series. No control
4. Bris et al. 2019	Non randomisation. Case series
5. Adamo et al. 2020	Non randomisation. Unavailable post treatment result for control
6. Jeong 2019	Follow up at 2 weeks
7. Iris et al. 2017	Follow up at 4 weeks
8. Ilankizhai et al. 2016	Review paper
9. Aravindhan et al. 2014	Review paper
10. Miziara et al. 2015	Review paper
11. Van Heerden WFP et al., 2011	Review paper
12. Garg et al. 2017	Non randomisation. No control. Case series
13. Jimson et al. 2015	Review paper
14. Skrinjar et al. 2020	Follow up at 2 weeks
15. Suga et al. 2019	Non randomisation. No control
16. Pereira et al. 2020	Review paper
17. Nakase et al. 2004	Non randomisation. Unavailable inclusion criteria on glossodynia . Follow up at 4 weeks
18. Bessho et al. 1998	Unclear definition on glossodynia. May included second burning mouth syndrome
19. Grechko et al. 1996	Non randomisation. Study included second burning mouth syndrome
20. Bardellini et al. 2019	Follow up at 4 and 5 weeks
21. Ritchie et al. 2018	Review paper
22. Barbosa et al. 2018	Follow up at 4 weeks
23. Sikora et al. 2018	Follow up at 2 weeks
24. De Souza et al. 2018	Systematic review paper
25. Liu et al. 2018	Systematic review paper
26. Fenelon M et al., 2017	Non randomisation. Retrospective study
27. Haggman-Henrikson et al. 2017	Systematic review paper
28. Kuten-Shorrer et al. 2017	Non randomisation. No control
29. Restivo et al. 2017	Non randomisation. Case series. No control
30. Al-Maweri et al. 2017	Systematic review paper
31. Valenzuela et al. 2017	Follow up at 2 and 4 weeks
32. McMillan et al. 2016	Systematic review paper
33. Sugaya et al. 2016	Follow up at 2 weeks
34. Cui et al. 2016	Systematic review paper
35. Valenzuela et al. 2016	Follow up at 30 days
36. Kisely et al. 2016	Systematic review paper
37. Arduino et al. 2016	Follow up at 21 days and 5 weeks
38. Treldal et al. 2016	Follow up at 2 weeks
39. Zakrzewska et al. 2016	Systematic review paper
40. Jurisic Kveisic et al. 2015	Follow up at 4 weeks
41. Lopez-Jornet et al. 2013	Control arm included active study treatment
42. Komiyama et al. 2013	No control group
43. Ko et al. 2012	Non randomisation. No control. Follow up at 4 weeks
44. De Moraes et al. 2012	Review paper
45. Silvestre et al. 2012	Follow up at 1 week
46. Buchanan et al. 2010	Review paper
47. Scardina et al. 2010	Non randomisation
48. Kho et al. 2010	Non randomisation. Follow up at 4 weeks.
49. Lopez-Jornet et al. 2010	Review paper
50. Gremeau-Richard et al. 2010	Non comparable follow up time. Clonazepam follow up at 3 weeks and local anaesthesia at 15 min
51. Barker et al. 2009	Non control. Same group of drugs in comparison
52. Miziara et al. 2009	Non comparative outcome assessment
53. Cavalcanti et al. 2009	Follow up at 30 days
54. Toida et al. 2009	Included secondary burning mouth syndrome patients
55. Buchanan et al. 2008	Review paper
56. Minguez Serra et al. 2007	Review paper
57. Patton et al. 2007	Systematic review paper
58. Buchanan et al. 2005	Review paper
59. Zakrzewska et al. 2005	Systematic review paper
60. Gremeau -Richard et al. 2004	Follow up at 2 weeks
61. Petruzzi et al. 2004	Non randomisation. Follow up at 30 days
62. Femiano et al. 2004	Non randomisation
63. Zakrzewska et al. 2003	Systematic review paper
64. Scala et al. 2003	Review paper
65. Femiano 2002	Unsure overlapping of recruited patient pools in Femiano 2000 trial or Femano and Scully 2002 trial
66. Maina et al. 2002	Included secondary burning mouth syndrome patients
67. Zakrzewska et al. 2001	Systematic review paper
68. Femiano et al. 2000	Follow up at 1 month
69. Sardella et al. 1999	Follow up at 4 weeks
70. Formaker et al. 1998	Non randomisation. No definition on burning mouth syndrome
71. Grushka et al. 1998	Non randomisation. No control
72. Dym et al. 2020	Review paper

### Characteristics of studies

All 22 included studies were randomised controlled clinical trials with one triple-blinded study (participant, caretaker, and assessor) (18), 14 double-blinded studies (19–32), four single-blinded studies (participants) (33–36), and three non-blinded studies (37–39). Three of the four single-blinded studies have a common concern with assessor blinding as they involved patient-reported outcomes (33,34,36). Fourteen (64%) studies described the method employed in generating the randomised sequence; online website or computer software, and randomisation tables, balls, or blocks (18–21,23–25,27–29,31,33,35,38). Eight studies reported on examiners’ allocation concealment (18,20,21,24,25,27–29). Five studies (22%) have a high risk of attrition bias (24,26,29,32,35), and eight studies (36%) have a high risk of reporting bias (20,22,24–27,31,32). In the reviewers’ opinion, none of the studies was graded high, with two very low (38,39), 12 low (22,24,26,27,30–37) and eight moderate (18–21,23,25,28,29).

Twenty studies were randomised controlled trials (RCT) with placebo parallel-group comparison (18–29,31–38), and two studies were a comparison between different parallel cohort treatment groups (30,39). The 20 placebo-controlled randomised trials consisted of 16 trials with two-arm (18–22,24,26–29,31,32,35–38) (14 intervention vs. placebo and two non-intervention vs. intervention), one trial with three-arm (23), and three trials with four arms (25,33,34) comparison between intervention and placebo. The remaining two non-placebo RCT were two-arm (30) and three-arm (39) trials investigating several different treatment interventions. Thirteen studies with a follow-up period between 2 and 3 months were categorised as short-term assessment (18,21–26,29,31–33,35,37). Seven studies were reporting long term assessments (> 3 months), ranging between 4 and 12 months (19,22,23,31,33,36,37).

The total pool of treated participants was 623, with a wide age range from 43 to 89 years. All BMS participants were appropriately defined as having chronic pain for more than 3 months, with normal oral mucosa and absence of contributing local or systemic factors, except De Rivera Campillo et al. (19) (duration of BMS was less than 6 months), Cinar et al. (39) (average duration of BMS was 17 days), Ottaviani et al. (31) (duration of pain was 1 month), and Bergdahl et al. (37) (no description of BMS duration).

The visual analogue scale (VAS) or visual numerical scale (VNS) of either 0–10 or 0–100 scores were the primary assessment tools in measuring post-therapy pain improvement (18,20,21,23,24,27–29,31,33–35,38) except Bergdahl et al. (37) with a VAS scale of 1–7. Six studies used categorical changes in pain improvement as their assessment tool (22,23,25,26,32,33). Supplementary assessment tools such as the McGill Pain Questionnaire (21,23,35,36), faces scales (29), Orofacial Pain Clinic Questionnaire (EDOF-HC) (32) and Brief pain Inventory (BPI) (35) were used to evaluate pain intensity and associated characteristics further. Face scales classified patients' expression of happiness based on a pictured face scale of 0–5 (lower is better). Secondary outcome assessment of participants' quality of health, anxiety and depression, and quality of sleep were evaluated using patient-reported questionnaires, such as 36-Short Form Health Survey (SF-36), Oral Health on Quality of Life (OHIP 14), Patient Health Questionnaires-9 (PHQ-9), Patient Global Impression of Change (PGIC), Clinical Global Impression for global Improvement Scale (CGC-Z), Hospital Anxiety and Depression Scale (HADS), Beck Depression Inventory (BDI), Zerssen Mood Scale (ZMS), Hamilton Rating Scale (HRS), Psychometric Symptom Checklist-90-R (SCL-90-R), Medical Outcomes Survey (MOS) of Sleep Scale and Epworth Sleepiness Scale (ESS).

The substantial heterogeneity in the treatment methodology and regime, the follow-up time and inadequately reported statistical data precluded formal meta-analysis on the efficacy of a treatment in this review. However, combined SMD VAS scores or RR of studies with similar interventions were pooled with 95% CI. Two studies without comparison with placebo (30,39) and another, which described outcomes using median values (27), were qualitatively analysed.

### Effects of treatment

**Figure 2.**
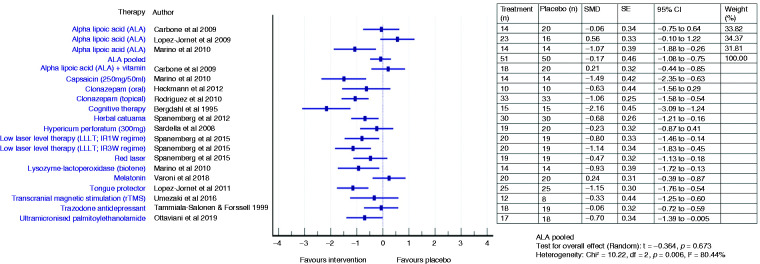
Forest plot showing standardised mean differences (SMD) and 95% confidence intervals for short-term outcomes (≥2 months and ≤3 months) of RCTs comparing an intervention with placebo for the treatment of BMS (with separate pooled effects for ALA).

**Figure 3. fig3-03331024211036152:**
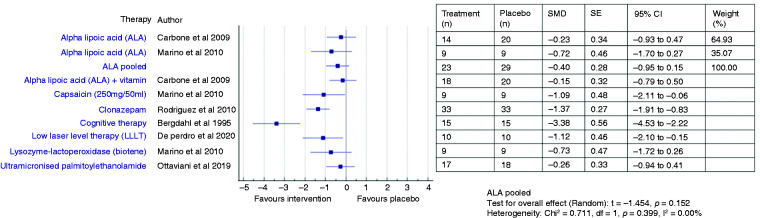
Forest plot showing standardised mean differences (SMD) and 95% confidence intervals for long-term outcomes (>3 months) of RCTs comparing an intervention with placebo for the treatment of BMS (with separate pooled effects for ALA).

**Figure 4. fig4-03331024211036152:**
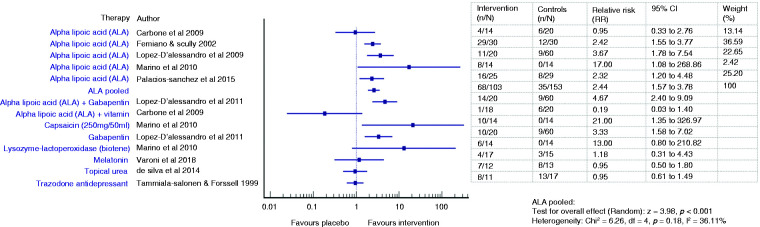
Forest plot showing relative risks (RRs) and 95% confidence intervals for short-term outcomes (improvement on VAS at ≤3 months) of RCTs comparing an intervention with placebo for the treatment of BMS (with pooled effect for ALA).

The effectiveness of various treatments and pooled efficacy for similar treatments for BMS between short- and long-term outcomes are shown in Figures 2 [Fig fig3-03331024211036152][Fig fig4-03331024211036152]to 5, respectively.

**Figure 5.**
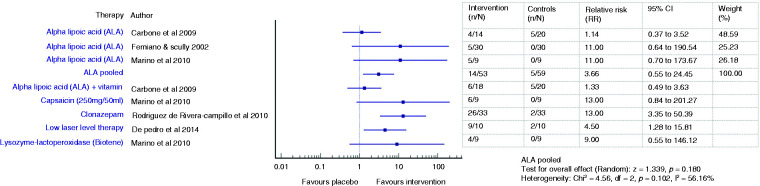
Forest plot showing relative risks (RRs) and 95% confidence intervals for long-term outcomes (improvement on VAS at >3 months) of RCTs comparing an intervention with placebo for the treatment of BMS (with pooled effect for ALA).

### Anticonvulsants

#### Clonazepam

The efficacy of clonazepam in reducing BMS pain symptoms was reported in two studies with oral (20,39) and one with topical administration (19).

##### Short term (2 months)

Treating BMS pain symptoms with daily oral systemic clonazepam 0.5 mg has shown favourable results of pain score reduction but was not statistically significant in the SMD analysis (SMD −0.63, 95% CI −1.56 to 0.29) (20). Despite the improvement in the taste, odour, and salivary flow rate, there were no statistically significant differences in improvement between clonazepam and placebo groups in taste (*p* = 0.83) and salivary flow (*p* = 0.03). Clonazepam did not improve patients’ ZMS mood and BDI depression scores.

##### Long term (4 months and 6 months)

Administration of 2 mg clonazepam has been reported to reduce VAS score significantly at 4 months (MD −4.1, *p* < 0.001) (39). Eight of the 25 participants developed side-effects such as dizziness (n = 4), transient diarrhoea (n = 2) and myalgia (n = 2) with the use of clonazepam. Within the clonazepam group, 70% of patients described an improvement in pain intensity, and three participants were completely asymptomatic after 6 months of daily rinsing with 0.5 to 2.0 mg clonazepam (19). The application of topical clonazepam significantly decreased patients’ VAS score (MD −4.7) (SMD −1.06, 95% CI −1.58 to −0.54) in comparison to placebo than oral ingestion clonazepam (20) (MD −3.2) (SMD −0.63, 95% CI −1.56 to 0.29) and no significant difference in the total number of tablets dissolved in the mouth as a topical application between both clonazepam and placebo groups. Six months of clonazepam rinse statistically significantly reduced pain scores by 13-fold (RR 13.0, 95% CI 3.35–50.39). Five clonazepam participants reported sleepiness as adverse effect, but they were not suspended from the trial.

#### Gabapentin

##### Short term (2 months)

Patients receiving 300 mg gabapentin has shown a similar result to alpha lipoic acid (ALA), with half of the total number of patients evidencing improvement in pain or total pain recovery (25). A more than three-fold likelihood of positive change relative to placebo were reported with the use of gabapentin in the short-term assessment of 20 BMS patients (25) (RR 3.33, 95% CI 1.58–7.02). It is associated with approximately a five-fold likelihood of decrease in pain levels compare with placebo if combined with ALA (RR 4.67, 95% CI 2.40–9.09) (25).

#### Pregabalin

##### Long term (4 months)

At 4 months of assessment, 150 mg pregabalin showed a significant reduction in VAS scores (MD −4.7, *p* < 0.001) (39). Six of the 25 participants had side effects such as increase in appetite (n = 3), vertigo (n = 1), mild nausea (n = 1) and diarrhoea (n = 1).

### Antidepressants

#### Trazodone

##### Short term (2 months)

Administration of 100 mg trazodone daily for the first 4 days followed by 200 mg for 8 weeks significantly decreased patients’ VNS pain intensity against baseline (MD −13.9, *p* < 0.01), but there was no significant difference with the placebo group (SMD −0.06, 95% CI −0.72 to 0.59; RR 0.95, 95% CI 0.61–1.49) (21). If the assessment was based on the “Patients’ Global Assessment of Improvement” evaluation, trazodone and placebo groups reported improvements in pain intensity of 73% and 76%, respectively, and were not significant (*p* > 0.05). One patient in the trazodone group reported a worsening of symptoms. Both the trazodone and placebo groups significantly improved their BDI depression scores (*p* < 0.01). The most common side effects were dizziness and drowsiness, with seven patients dropping out due to dizziness. Other side effects included abdominal pains, headache, palpitation, tremor, xerostomia, and urinary incontinence.

#### Citalopram

##### Short term (11 weeks)

The use of citalopram 10 mg daily followed by an increment to 20 mg after 1 week showed an improvement of VAS score of 87.45% (MD: −7.8, *p* < 0.001) (30). However, comparison with crocin reported no significant difference between their post treatment VAS scores (*p* = 0.98). The Hamilton questionnaires analysis revealed a significant reduction of depression and anxiety scores, with an average recovery percentage of improvement of 30.57% (SD 15.81) and 15.44% (SD 11.86), respectively. There was no significant difference in comparison between both groups in depression (citalopram: 19.4, SD 4.65; crocin: 19.0, SD 3.97, *p* = 0.76) or anxiety (citalopram: 18.6, SD 5.11; crocin: 18.0, SD 4.38, *p* = 0.76).

### Phytomedicine

#### Topical capsaicin

##### Short term (2 months)

Rinsing with 250 mg of chilli powder emulsified in 50 ml water with a dose concentration of 3.54 µg⁄ml capsaicin has been reported to induce a significant reduction in VAS score (MD −3.2, *p* < 0.01) with 76% of participants reporting an improvement in symptoms, but one patient-reporting a worsening (33). Capsaicin provides an immediate short term pain relief (SMD −1.49, 95% CI −2.35 to −0.63) and is statistically significant with 21 times better than placebo (RR 21.00, 95% CI 1.35 to 326.97). Topical capsaicin has shown a better clinical pain management outcome than oral ALA and lysozyme lactoperoxidase, despite no statistically significant VAS difference in intergroup comparison.

##### Long term (4 months)

Capsaicin showed superiority in maintaining VAS score reduction in long term (MD −2.9, *p* = 0.03) compared to lysozyme-lactoperoxidase, boric acid rinse and ALA (33). It also demonstrates sustainable benefit in long term administration (SMD −1.09, 95% CI −2.11 to −0.06) (33). It is 13 times better than placebo but not statistically significant (RR 13.00, 95% CI 0.84–201.27). An improvement in pain intensity was reported by 67% of participants, while one patient remained the same, reported worsening of pain. No adverse effect was noted during the trial.

#### Ultramicronised palmitoylethanolamide (umPEA)

##### Short term (2 months) and long term (4 months)

Ottaviani et al. revealed a short-term (60 days) benefit with 1200 mg/day umPEA in BMS patients (SMD −0.70, 95% CI −1.39 to −0.01) but declining pain relief at 4 months (SMD −0.26, 95% CI −0.94 to 0.41) compared to placebo group (31). There were no side effects observed in patients treated with umPEA.

#### Herbal catuama

##### Short term (3 months)

Catuama shows promising VNS (0–10) score reduction results compared to placebo with a minimal adverse effect of sleep alteration observed in the study (SMD −0.68, 95% CI −1.21 to −0.16) (29). Catuama shows a greater alleviation of patient symptoms with a lower faces scale score at both 8 and 12 weeks than placebo (*p* ≤ 0.001). The mean reduction of the face score were 1.6 and 1.5 for 8 and 12 weeks, respectively, while there were no changes in participants’ happiness in the control group with a similar mean reduction faces scale scores of 0.6 at 8 and 12 weeks. The majority of patients tolerated the treatment well, with none of the patients in the test group reporting xerostomia. The side effects reported by patients that took Catuama included somnolence and weight gain (n = 1), insomnia (n = 1), and exacerbation of the pain symptoms intensity in the first week of treatment (n = 2). A drop-out of eight (21.1%) participants in the treatment group, and four (11.8%) in the placebo group were reported.

#### Hypericum perforatum

##### Short term (3 months)

At the end of 12 weeks of therapy, there was a reduction in the number of oral mucosa burning sites and improved ability to cope with the burning pain, there was no statistically significant difference with the placebo group (SMD −0.23, 95% CI −0.87 to 0.41) (28). The HAD questionnaires showed that approximately 50% of patients in both treatment and placebo groups evidenced better coping ability on their pain symptoms at the end of the trial. One participant developed a severe headache in the fifth week of active therapy (28).

#### Crocin

##### Short term (11 weeks)

Crocin showed a significant reduction in VAS score (MD −7.8, *p* < 0.001) and has a similar improvement 87.5% of burning mouth score as citalopram (30). A significant improvement in depression and anxiety scores by 30.79% (SD 13.24) and 15.40% (SD 13.98), respectively, were reported. Crocin displayed similar effects as citalopram in treating burning pain, depression, and anxiety.

#### Lycopene enriched extra virgin oil (LVO)

##### Short term (3 months)

A combination of topical spray and swallowing of 900 ppm LVO daily for 12 weeks led to a significant reduction in the median pain score (MD −3.0, *p* < 0.001) and burning (MD −1.0, *p* = 0.003) compared to baseline, but there was no significant difference (*p* = 0.99) when compared with the placebo group (27). Evaluation of SP-36 and OHIP-14 questionnaire scores showed no difference in changes to quality of life between treatment and placebo groups. HAD anxiety scores did not differ between treatment and placebo groups or significantly change throughout the trial period. The cholesterol and triglycerides levels were not remarkably raised after 12 weeks of LVO administration.

### Alpha lipoic acid (ALA)

#### Short term (2 months)

Four ALA trials (22,25,26,33) showed promising pain reduction in comparison to placebo during short term assessment (Femiano and Scully: RR 2.42, 95% CI 1.55 to 3.77; Lopez D’alessandro: RR 3.67, 95% CI 1.78 to 7.54; Palacios-Sanchez: RR 2.32, 95% CI 1.20 to 4.48; Marino: RR 17.0, 95% CI 1.08 to 268.86) while two did not (Carbone: SMD −0.06, 95% CI −0.75 to 0.64; RR 0.95, 95% CI 0.33 to 2.76; Lopez Jornet: SMD 0.56, 95% CI −0.10 to 1.22) (23,24). The pooled ALA suggested a more than double increase in likelihood of pain improvement (RR 2.44, 95% CI 1.57 to 3.78, *p* < 0.001) compared to placebo (22,23,25,26,33). However, there were no significant changes in the pooled ALA VAS scores (SMD −0.17, 95% CI −1.08 to 0.75, t −0.36, *p* = 0.72), reflecting the heterogeneity across studies (23,24,33). One patient had to discontinue treatment during the trial due to gastrointestinal upset such as nausea, dyspepsia and pyrosis (24). 

#### Long term (4 months and 12 months)

Two studies (23,33) assessed the persistence of the observed improvement for 2 months after discontinuation of therapy and described a stable decrease of VAS score (Carbone: MD −1.8, SD 3.19, *p* = 0.01; Marino: MD −1.8, *p* > 0.05). Long-term use of ALA did not result in any statistically significant improvement over placebo, suggested by the pooled VAS mean score changes (SMD −0.40, 95% CI −0.95 to 0.15, *p* = 0.15) (23,33) and the likelihood of improvement (RR 3.66, 95% CI 0.55–24.45, *p* = 0.18) (22,23,33).

A study comparing ALA 600 mg with two other drugs (clonazepam and pregabalin) showed no significant improvement at 4 months of assessment (MD −0.72, *p* > 0.05). Three out of 25 patients reported side effects, including mild nausea (n = 2) and myalgia (n = 1) (39). A 1-year follow-up showed a sustained effect on pain intensity in 73% of patients. In this study, patients with signs of improvement within the first 4 months of treatment were given an extended treatment of 1 month ALA 600 mg (22).

#### ALA and gabapentin

##### Short term (2 months)

A combination of 600 mg ALA and 300 mg gabapentin in a randomised, double-blind clinical trial described a notable pain reduction, with 70% of patients demonstrating a partial or complete improvement in pain intensity compared to 15% in the placebo group (25). The combined use of ALA and gabapentin gave a five-fold likelihood (RR 4.67, 95% CI 2.40–9.09) (*p* < 0.001) of decrease pain intensity while ALA only has four times the likelihood of beneficial effect (RR 3.67, 95% CI 1.78 to 7.54).

#### ALA and vitamins

##### Short term (2 months) and long term (4 months)

Combining vitamins such as vitamin C, PP, E, B6, 2,1, 12 and folic acid with 800 mg ALA did significantly improve VAS score (MD −1.0, SD 1.83, *p* = 0.047) and a further reduction in VAS score was noted 2 months after termination of treatment (MD −1.8, SD 3.19, *p* = 0.047) (23). However, there was no significant difference between ALA and vitamins (SMD 0.21, 95% CI 0.44–0.85) (SMD −0.15, 95%CI −0.79 to 0.50) compared to ALA monotherapy (SMD −0.06, 95%CI −0.75 to 0.64) (SMD −0.23, 95%CI −0.93 to 0.47) or placebo in both short (*p* = 0.60) and long-term assessment (0.79). ALA as a monotherapy led to a higher reduction in VAS score at 2 months (MD −1.6, *p* = 0.013) but no statistically significant difference compared to placebo (*p* = 0.60) compared to baseline, but there was no significant difference between the ALA (monotherapy), ALA and vitamin (combination) and placebo groups. No adverse effects were reported in the study (23).

### Melatonin

#### Short term (2 months)

A cross-over clinical trial involving intervention with a high melatonin dosage (12 mg/day) did not provide pain relief (SMD 0.24, 95% CI −0.39 to 0.87; RR 1.18, 95% CI 0.31–4.43) and sleep score improvement compared to placebo (18). Ten participants reported no changes in symptoms, and one participant reported worsening of symptoms. The value of VAS score and serum plasma melatonin concentration was negatively associated, but it was not statistically significant (*p* > 0.05). Two patients in the melatonin group demonstrated a positive correlation between decreased VAS scores and increased sleep hours. The Hamilton rating scale for anxiety (HAM) assessment scores was always higher in the melatonin group than placebo, with a statistically significant decrease in the melatonin group’s anxiety score (*p* < 0.05). An approximate two-fold of patients reported sleep impairment using melatonin (n = 10, 62.5%) compared to placebo (n = 6, 37.5%). Mild daytime sleepiness was seen in melatonin and placebo groups, with high ESS scores but not significant between them (p > 0.05). The main adverse effect of melatonin that leads to the discontinuation of treatment on four patients were heavy tremor, sexual disturbances, blurred vision, and severe heavy headedness. Four patients were dropped from the study due to lack of efficacy, pain improvement, and follow-up loss.

### Low-level laser therapy (LLLT)

#### Short term (11 weeks)

A significant reduction in pain score by three to five units was observed in the study using the red (*p* = 0.13) and infrared laser (IR1W *p* = 0.004 and IR3W *p* < 0.001) (34). The red laser group (SMD −0.47, 95% CI −1.13 to 0.18) did not demonstrate a significant difference from the control group, but both IRW1 (SMD −0.80, 95% CI −1.46 to −0.14) and IRW3 (SMD −1.14, 95% CI −1.83 to −0.45) showed a statistically significant difference from the control group (34). No side effects were noted from the laser therapy.

#### Long term (4 months)

A recent trial has suggested the advantage of photobiomodulation in treating orofacial neuropathic pain, including BMS with a significant 4.5-fold likelihood of pain reduction in comparison to placebo (RR 4.50, 95% CI 1.28–15.81) and a more than 1-point decrease in VAS (SMD −1.12, 95% CI −2.10 to −0.15) (36), but no improvement in patients’ psychology and quality of life. There was no significant improvement in McGill Pain scores, patient oral health quality scores (OHIP), physical and emotional scores (SF-36) and sleepiness (ESS). However, there was a significant decrease in SCL-90-R interpersonal sensitivity, somatisation, and anxiety between the photobiomodulation group and the placebo group (*p* = 0.04). No adverse effects were reported.

### Saliva substitutes

#### Topical lysozyme lactoperoxidase (Biotene)

##### Short term (2 months) and long term (4 months)

Lysozyme lactoperoxidase (Biotene) rinse was prescribed to BMS patients diagnosed with xerostomia (33) and reported a decrease in pain score of 1.7 units during short-term assessment (SMD −0.93, 95% CI −1.72 to −0.13) but no advantage over placebo was seen in long-term assessment (SMD −0.73, 95% CI −1.72 to 0.26). A 13-fold (RR 13.00, 95% CI 0.80–210.82) and nine-fold (RR 9.00, 95% CI 0.55–146.12) likelihood of pain reduction compared with placebo was observed in both short- and long-term analyses (33).

The lubricating rinse lysozyme lactoperoxidase significantly reduced the VAS score (MD −1.7, *p* = 0.01), but there was no significant difference between lysozyme lactoperoxidase with capsaicin rinse and oral ALA, respectively (33). The pain score remained unchanged in 57% and 55% of patients in both short and long-term assessment.

#### Topical urea

##### Short term (3 months)

Statistical analysis showed no statistically significant difference between the application of 10% urea for 3 months and the placebo group (*p* = 0.34) (RR 0.95, 95% CI 0.50–1.80) (32). There is no difference in pain intensity after treatment (*p* = 0.88), although clinically 58.3% of patients demonstrated a reduction in pain intensity.

### Transcranial magnetic stimulation (rTMS)

#### Short term (2 months)

Ten days of 30,000 pulses of rTMS therapy over the left GDLPFC significant reduced VAS score (MD: −3.1, *p* = 0.002) with 75% of patients reporting a decrease in pain intensity of more than 50% compared to baseline (35). There was a significant difference compared with placebo (MD: −2.8, *p* = 0.005) (SMD −0.33, 95% CI −1.25 to 0.60). There was a significant improvement in sensory SFMPQ in the rTMS group (MD −4.84, *p* = 0.002) but no difference in the SFMPQ affective scores and present pain intensity. PGIC and CGO-I assessments described positive changes from the patient in the rTMS group. There were no significant changes in patient mood based on PHQ-9 (MD 5.59, *p* = 1.00).

### Tongue protector

#### Short term (2 months)

The hypothesis of wearing the tongue protector to prevent continuous irritation of tongue on teeth or denture has a statistically significant difference in improvement in VAS score between wearer (MD −3.6) and non-wearer with habitual avoidance reminder (MD −1.4, *p* < 0.001; SMD −1.15, 95% CI −1.76 to −0.54) (38). Participants did not show any improvement in the depression and anxiety score. There was a significant improvement in patient quality of life based on OHIP-49 and SF36 assessments.

### Cognitive therapy

#### Short term (12–15 weeks) and long term (6 months)

At the end of weekly behavioural therapy for 12–15 weeks, patients reported a significant improvement in their pain score for both short- (SMD −2.16, 95% CI −3.09 to −1.24) and the long-term effects were sustained over 6 months post-treatment: (SMD −3.38, 95% CI −4.53 to −2.23) (37). There were statistically significant changes between the therapy and the placebo group (*p* < 0.001).

## Discussion

At present, there is no definitive curable treatment for BMS. Its aetiology remains uncertain with various suggested pathogenesis such as peripheral and central neuropathy disorders, psychological disorders, changes in gonadal, adrenal and neurosteroid levels, a dopamine D2 receptor (*DRD2*) 957C>T genotype and the association between BMS and other neurological diseases such as Parkinson’s disease (40–43). BMS treatment primarily aims at eliminating the painful burning dysaesthesia. Phenotyping BMS patients’ aetiology could achieve this based on their clinical histories and responses toward various treatments. In this review, we discuss nine BMS therapies: Anticonvulsants (19,20,25,39), antidepressants (21,30), phytomedicines and food supplements (18,22–29,31–33), lower-level laser therapy (34,36), saliva substitute (32,33), transcranial magnetic stimulation (35), oral appliances (38) and cognitive behavioural therapy (37).

Preceding systematic reviews included clinical trials of 2 weeks follow-up assessment results. It is crucial to have a more extended review period of patients’ responses towards the therapy, the sustainability of the treatment effects and the possible side effects before considering that a treatment has been effective. Hence, to ensure sufficient, sustainable benefits of the treatments, this review includes studies with a minimum follow up of 2 months and divided them into short term (≤ 3 months) and long term (> 3 months) treatments (11).

The majority of the included studies had small sample sizes. The diversified BMS patients’ characteristics such as presence or absence of psychological disorders, taste disturbance, and xerostomia make recruitment for a larger homogenous sample group difficult in a clinical trial. The concurrent use of psychotherapeutic drugs or therapies and anti-inflammatory analgesic medications in patients may influence the presentation of the BMS population trials due to the ambiguity of whether these psychological disorders preceded BMS (21,26,32,35,38).

### Anticonvulsant

#### Clonazepam

Both oral ingestion and topical application of clonazepam have showed a favourable result on BMS pain relief up from 2–6 months (19,20,39). The association of the peripheral or central nervous system in BMS pathogenesis explained the use of antiepileptic and antidepressant drugs. Continuous nociceptive peripheral neuropathy input will eventually lead to central sensitisation and changes. Pharmacological drugs such as clonazepam demonstrated their analgesic ability by inhibiting neurological transduction and transmitting the pain signal. Clonazepam, a benzodiazepine anticonvulsant drug, acts as an agonist modulator on GABA-A receptors and activates the descending pain inhibitory pathway of the peripheral (PNS) and central nervous system (CNS) by facilitating the opening of the chloride channel. It antagonises the neuron hyperexcitability transmission by generating a continuous hyperpolarisation, thus preventing depolarisation and post deafferentation neuronal firings (44). GABA-A receptors are found in the oral mucosa, mandible, palate, salivary gland, and taste pathway. GABA agonist could reverse the dysfunction of peripheral chorda tympani nerve and taste loss in BMS patients (45). Clonazepam could provide fast and continuous pain relief due to its rapid absorption and 90% bioavailability of clonazepam within 1–4 h after oral administration and its long half-life of 30–40 h.

Meanwhile, intraoral topical clonazepam has shown to be superior to oral ingestion in providing much rapid pain analgesia but a shorter duration of action. Patients reported rapid positive effects within 10 min upon dissolving the clonazepam tablet intraorally and recurrence of pain in 3–4 hours (19). The topical clonazepam route is simple with a rapid and shorter duration of action, which allows repetitive use and lower risk of common systemic adverse effects such as drowsiness, dizziness, and unsteadiness. It allows patients to have better self-control over pain relief magnitude in their daily activities. Inevitably, some of the topical clonazepam will be absorbed systemically through the oral mucosa and affect the CNS pain modulation. This is reported in a study assessing patient’s post topical clonazepam serum concentration, which was similar between 5 h post sucking a 1mg clonazepam tablet and after sucking the tablets three times daily for 14 days (46).

The use of amitriptyline, a tricyclic antidepressant, commonly used to treat chronic neuropathic pain, has not been widely mentioned in BMS studies. This may be the result of the frequent xerostomia induced by amitriptyline, whiich aggravates the pre-existing BMS-related xerostomia. A retrospective study has reported a superior rapid decrease of VAS pain scores outcome for clonazepam drops (n = 23) compared to amitriptyline drops (n = 16) at 6 weeks but no statistical difference between them (47).

#### Gabapentin and pregabalin

Gabapentin and pregabalin have been the favourable drug choice in treating neuropathic pain conditions such as diabetic neuropathy and postherpetic neuralgia due to its hepatic safety profile (48). Similar advantages in BMS pain were achieved with the use of gabapentin and pregabalin in short- and long-term assessment (25,39). Gabapentin mediates pain attenuation by binding to the α2δ-1 subunit of the voltage calcium channels and inhibits the release of neurotransmitters such as glutamate, CGRP and substance P; the development of chronic pain (49,50) correlates BMS as a neuropathic pain that may involve both central and peripheral mechanisms. The benefits of gabapentin in BMS with peripheral neuropathy disorders may suggest using adjunct dietary supplements such as ALA to enhance the pain attenuation without increasing the synthetic drug's needs. However, a more extensive sample size study is recommended to test the efficacy of gabapentin and its adverse effects. Cinar et al. compared the use of systemic pregabalin (150 mg) with clonazepam (2 mg), and both drugs show similar significant efficacy in reducing pain score (39). A third of patients in both study groups had common adverse effects, but no patients withdrew from the study. The absence of a placebo group in the study failed to give a definitive superiority outcome between pregabalin and clonazepam (39).

### Antidepressants

BMS has been strongly associated with depression and anxiety, and the lack of clarity between them is unsettling. This neurophysiological mechanism in BMS was shown in functional magnetic resonance imaging (fMRI) (51) and quantitative somatosensory testing (QST) study (52). fMRI study has reported an increase in the region’s functional neural activity regulating depression and anxiety in BMS patients (51). It is known that chronic anxiety and depression may disturb neuroprotective steroid productions (53). As pain could be a somatic trait, the use of an antidepressant has suggested the role of anxiety and depression in BMS pathogenesis.

#### Trazodone

Trazodone is a second-generation antidepressant that has been considered a multifunctional drug and acts as a serotonin reuptake inhibitor. Trazodone has been used in treating anxiety and pain symptoms, including fibromyalgia (54). However, in this review, trazodone use did not significantly affect pain reduction and had a high placebo effect. The reported high adverse effects on dizziness and drowsiness limit its use (21).

#### Citalopram

Citalopram has shown to be able to reduce pain intensity (30). A review of SSRIs such as zimelidine, sertraline, citalopram, paroxetine, and fluoxetine has suggested it for the treatment of chronic pain conditions (55). The SSRI citalopram has similar antidepressant and analgesic properties to tricyclic antidepressants but with significantly fewer side effects and better tolerability (56). Serotonin is a neurotransmitter that plays a role in both central and peripheral nociception and mood regulation. SSRIs inhibit serotonin’s reuptake and prolong its availability in the synaptic cleft. There was inconclusive effectiveness in treating chronic pain with SSRIs. Inconclusive results were observed from various studies on its use for chronic somatoform pain and fibromyalgia. As there is no placebo group in comparing the efficacy of citalopram in reducing burning mouth and less than 50% of patients recovered from depression and anxiety, there is limited evidence to support its use (30). Clinical trials with better methodology and low risk bias are needed to conclude the effect of SSRI as a treatment for chronic pain conditions.

### Phytomedicine

The perspective of using herbal medicine or phytomedicine has been established and increased in primary health care (57). The efficacy of phytomedicines such as capsaicin, herbal catuama, umPEA and hypericum perforatum have demonstrated their analgesia ability, with capsaicin having a tremendous number of patients in responding to it. Through well-designed randomised control trials and observational studies, phytomedicine has a tremendous future to be used solely or as adjunct therapy in treatment therapeutic strategies and products (58).

#### Capsaicin

Capsaicin has shown to be an effective pain desensitiser especially with oral topical application for up to 4 months (33). Transient receptor potential vanilloid-1 receptors (TRPV1) are found in the PNS and CNS (59). The numbers of TRPV1 receptors are significantly increased in the mucosa of BMS patients’ tongues (60). Activation of TRPV1 at the peripheral terminal fibre endings leads to the release of neuropeptides such as substance P, neurokinin A (NKA) and calcitonin-gene-related peptide (CGRP), which contributes to the onset of hyperalgesia pain and inflammation. Local capsaicin application activates the TRPV1 and modulates the nociceptive transmission of pain impulses from the peripheral stimulation site to the central nervous system by blocking axonal transportation, depleting neuropeptides, and loss of membrane action potential. Hence, capsaicin-induced analgesic effect by desensitisation of the nociceptive fibre (61–62), which is a reversible process (63). The used of topical capsaicin have been suggested in neuropathic pain, such as postherpetic neuralgia and painful HIV associated polyneuropathy (64–66) but not inflammatory pain such as osteoarthritis (67).

A study showed no difference between systemic and topical capsaicin efficacy in BMS (68). However, gastric pain limits systemic capsaicin use (68). The use of topical capsaicin rinse is recommended in BMS due to its rapid action and there being no reported adverse effects, as seen in other synthetic drugs. However, there are no known risks of long-term repeated rinsing of capsaicin, especially in the oral cavity mucosa innervation. Patients should be warned of the initial increase in burning pain induced by topical capsaicin rinse or application followed by the discharge in the C and Aδ nociceptive fibres, but this effect is limited, of short duration, and followed by pain relief. Cutaneous site pre-treatment with anaesthetic cream has been used clinically to reduce the capsaicin patch-induced treatment discomfort in patients with peripheral neuropathic pain (69). Hence, a possible hypothetical proposition of a mouth rinse mixture containing both capsaicin and lidocaine may mask this initial burning pain and enhance pain relief effectiveness.

#### Ultramicronised palmitoylethanolamide (umPEA)

There is a small reduction of pain score with umPEA but its effect did not sustain (31). Systemic administration of PEA elicits anti-inflammatory, antinociceptive, and neuroprotective effects, both *in vivo* and *in vitro* (70,71), as well as in human subjects (72,73). Neurodegeneration could occur due to inflammatory reactions and activation of immune cells. Microglia facilitates the CNS’s inflammatory response, and white mast cells coordinate PNS inflammation. umPEA is an endogenous fatty acid that suppresses the discharge of proinflammatory mediators from mast cells and microglia during inflammation, thus preventing neuronal injury and chronic pain. A meta-analysis study has reported umPEA as a novel treatment in managing chronic neuropathic pain caused by neuroinflammation (74). A study of 40 days umPEA has reported positive benefit in diabetic or traumatic peripheral neuropathic pain (75). The novelty of umPEA efficacy as a primary or adjunct treatment in BMS should be further studied with a larger cohort and follow-up period for its sustainability.

#### Herbal catuama

Three months used of catuama has shown a significant reduction in BMS pain score (29). Catuama is a herb commonly used for mental and physical exhaustion. It has been shown to have antidepressant, antinociceptive and vasorelaxant actions in animal models by acting on the dopaminergic, serotoninergic, and opioid pathways and reducing the inflammatory nociception in animal models (76). It is thought that catuama may alleviate the burning pain based on the possible BMS aetiologies of psychologic and neuropathic disorders. A more extended observation on the use of catuama is suggested to ensure its long-term adverse effects and suitability as a pain relief.

#### Hypericum perforatum

The short-term use of hypericum perforatum in BMS has shown a favourable outcome but not significantly better than placebo (28). Hypericum perforatum (St. John’s Wort extracts) has been used as an antidepressant in mild to moderate depression, anxiety and sleep disorders (77) and may be beneficial to BMS patients as they frequently experience emotional and mood distress, in which anxiety and depression could be the primary or secondary event. Several active extracts in hypericum perforatum have a strong affinity for γ-aminobutyric acid (GABA), adenosine, serotonin 5HT_1_ as well as benzodiazepine receptors, and act as monoamine oxidase inhibitors (MAOI) (78). Its action as a MAOI prevents the reuptake of norepinephrine, serotonin and dopamine neurotransmitters from the brain, providing beneficial antidepressant effects. As a GABA agonist, it induces a temporary hyperpolarisation of the neuronal membrane and ensuing desensitisation and inhibition of neurotransmission, which provides an anxiolytic and analgesic effect (79).

Hypericum perforatum rarely causes any adverse drug reactions, except for dizziness, and is usually well tolerated by the elderly (80). It has comparable efficacy and safety compared to SSRIs in patients with mild to moderate depression (81). However, there is inadequate evidence on its long-term efficacy and safety, especially in patients with severe depression or suicidal risk.

Although it is relatively safe, clinicians should be wary of prescribing hypericum perforatum with other medications as it may elicit severe clinical adverse drug interaction effects. Hypericum perforatum activates the cytochrome P450 enzymes involved in drug metabolism, and reduces the plasma concentration and potency of a number of drugs such as warfarin (risk of thrombosis), cyclosporin (risk of transplant rejection), oral contraceptives (unintended pregnancy), anticonvulsant (uncontrolled seizures), digoxin (cardiac arrhythmia), theophylline (poor asthmatic control), and HIV protease inhibitors and non-nucleoside reverse transcriptase inhibitors (diminution in HIV suppression) (82). Caution should also be taken in combining hypericum perforatum with medications that have serotoninergic effects as it increases the serotoninergic action of serotonin receptor agonists (triptans) as well as of selective serotonin reuptake inhibitors (SSRI), selective norepinephrine reuptake inhibitors (SNRIs), tricyclic antidepressants (TCAs) and monoamine oxidase inhibitors (MAOIs) (82,83).

#### Crocin

Crocin is a carotenoid chemical compound found in the flowers crocus and gardenia and is responsible for the colour of saffron. Crocin prevents neuroinflammation and neurodegeneration by decreasing oxidative stress and cell death (84) by inhibiting microglial activation and suppressing inflammatory cytokine production (85). Microglia dysfunction contributes to the disturbance in their protective regulator function on neuroinflammation stimuli and generates an imbalance of reactive oxygen species (ROS) homeostasis and the antioxidant system, creating oxidative stress (86,87). Oxidative stress is associated with neurodegeneration through several cascades of deleterious events on the cells, causing lipid peroxidation, protein oxidation and mitochondrial DNA damage, and mutations (88). The accumulated increased oxidative stress in the aged brain has been thought to be a possible aetiology of neurodegenerative diseases such as Parkinson’s disease and Alzheimer’s disease. There have been reports on BMS occurrence in a patient with Parkinson’s disease (89,90), but there is no study on dysfunction of microglia and mitochondria and the oxidative stress in BMS patients. The brain is much more vulnerable to this oxidative stress due to its high oxygen demand and lipids’ vital role in maintaining neuronal function (91). Neuroprotective effects of crocin have been shown in an experimental animal model (84), but not in more extensive human clinical trials on its long-term safety and benefits. This review shows a significant improvement in crocin pain score but no significant superiority over citalopram (30). A three-arm- study design with placebo control group comparison is advised to compare crocin and citalopram's superiority.

#### Lycopene and virgin olive oil (VOO)

Lycopene is naturally found in red carotenoid pigmented food, such as in tomatoes. It has antioxidant, anti-inflammatory, and anti-apoptotic properties. These benefits have been seen in reducing cancer and cardiovascular risk with the consumption of lycopene and VOO (92,93). Combination of lycopene and VOO is thought to provide a synergistic effect of antioxidative and anti-inflammatory mechanisms. The ingestion of lycopene with olive oil will increase bioavailability (94). The application of topical lycopene and VOO may protect the oral mucosa’s peripheral neurons from oxidative stress, while VOO provides a lubricant effect. However, lycopene and VOO are not superior to placebo in improving pain score and health quality (27).

### Alpha lipoic acid (ALA)

ALA is the most studied treatment in BMS. Although the VAS findings from the pooled ALA analysis suggested there was no significant reduction in pain intensity relative to placebo treatment, a significantly higher proportion of patients reported pain reduction with ALA. As such, it suggests ALA as a treatment for BMS, but the evidence is not conclusive due to the variability of the studies treatment regimens and short- and long-term study results (9–11).

ALA is a naturally occurring compound found in the body and vegetables such as tomatoes, potatoes, broccoli, and brussels sprouts. It acts as an enzymatic cofactor for pyruvate dehydrogenase and α-ketoglutarate dehydrogenase complexes in glucose and lipid metabolism. ALA is a robust universal antioxidant and can chelate and remove heavy metals from the body. Thus, it reduces oxidative stress-induced inflammation and damage to the nerve. ALA's advantages and safety were demonstrated in the treatment of diabetic polyneuropathy pain and paraesthesia by preventing nerve fibre degeneration (95,96). Hence, the possible goal of administering ALA in BMS patients is to treat patients with peripheral neuropathy as the pathogenesis. The bioavailability of oral ALA is strongly affected by its formulation and its regime due to its reduced solubility and stomach instability. ALA in liquid form is preferred over solid for better absorption and should be taken pre-meal. Age influences the bioavailability of ALA. Patients aged above 75 years have better absorption rates than 18 and 45 years, but there was no difference in gender (97). As BMS commonly occurs in the fifth to seventh decade of age, ALA may be a beneficial adjunct supplement to ease the pain. In this review, the mean age reported ranged between 45 to 67 years.

ALA and gabapentin have shown a superior result, with mild adverse effects reported (25). Combined ALA use as an adjunct supplement to pharmacotrophic drugs may benefit the patients in minimising the drug’s adverse effects by reducing the prescribed frequency and dosage. However, studies with larger sample sizes and longer follow-ups of a minimum of 6 months with better methodology design should be conducted to validate the use of ALA.

### Melatonin

There was insufficient evidence of the benefit of melatonin in BMS. The relationship between pain and sleep is inextricable, in which poor sleep quality is a risk factor for chronic pain development, and pain disrupts the sleep pattern (98). Melatonin is a neurohormone that regulates the circadian biological rhythms. Melatonin has antioxidant, anti-inflammatory, anticancer, anxiolytic and antinociceptive activities (99). It has been shown to reduce chronic pain in fibromyalgia (100) and temporomandibular joint disorders (101). The analgesic effect of melatonin in neuropathic pain has been demonstrated in animal models (102,103). The use of exogenous melatonin in neuropathic pain is controversial due to multiple complex analgesic mechanistic pathways (104). A notable 40% drop-out rate was seen using melatonin due to heavy tremor, sexual disturbances, blurred vision, and heavy-headedness (18), despite the claim that melatonin is well tolerated and safe at high doses (105). As sleep disturbances are uncommon in BMS patients, this may in part explain the poor treatment response of BMS-related pain to melatonin.

### Low-level laser therapy

Photobiomodulation with low-level laser therapy (LLLT) effectively reduces chronic pain such as low back pain, temporomandibular joint disorder, and osteoarthritis (106). LLLT facilitate analgesia via its anti-inflammatory effects by increasing the secretion of serotonin, endorphins and adenosine triphosphate, augmentation of the cell membrane potential and suppressing impulse conduction velocity (107). The infrared laser has a longer wavelength compared to the red laser. It will penetrate tissue deeper, reaching the nerve fibres (108). This is observed in Spanemberg et al., where the infrared laser has a higher, significant difference in the reduction of pain score compared to placebo, but the red laser showed no difference to the control group (34). Increasing the intensity of the laser therapy application has remarkably augmented the significance of pain score improvement compared to placebo as seen in IRW3 with three sessions per week compared to IRW1 with one session a week. In summary, LLLT seems to be able to contribute to BMS patients’ pain relief and the possibility of being used along with pharmacological and psychological treatment for a better outcome. The beneficial effect of LLT is sustained from 1–4 months after 10 sessions of LLLT (36). It is suitable for use in medically compromised or patients on polymedication for pain as it is a non-invasive technique with no known reported adverse effects.

### Saliva substitute – Biotene and urea

BMS patients often complain of dry mouth discomfort (109). The lower salivary flow rate and thicker saliva froth may disturb the taste function (110). Urea and lysozyme lactoperoxidase (Biotene) are topical anti-xerostomic medication (saliva replacement). De Silva et al. studied urea as an adjunct therapy in BMS patients who were concurrently treated with amitriptyline (32). Amitriptyline is the first line of drug used in treating chronic neuropathic pain (111) and is known to cause dry mouth. There was no beneficial improvement seen in burning pain, taste and somatosensory despite increased oral cavity moisture and lubrication with urea or Biotene. BMS patients have decreased unstimulated salivary flow rate but not stimulated saliva. There was no objective hyposalivation observed, which explain the lack of oral cavity lubricants efficacy in reducing the pain intensity (110,112) and the possibility of central neuropathy as the pathogenesis. Caution should be taken due to the small participant size of less than 20 in both studies (32,33).

Anecdotal patient claims suggest regular sips of ice water help elevate the pain, which may be due to stimulation of transient receptor potential melastatin 8 (TRPM8) cold receptors or antagonist effect on TRPV1 found in the oral mucosa. The role of TRPM8 in pain analgesia has been widely contradictory debated, which may depend on its anatomical site and degree of activation (113).

### Transcranial magnetic stimulation (rTMS)

Neuroimaging studies have demonstrated BMS patients to have similar brain pain matrix changes with increased functional connectivity and reduced grey matter volume as seen in other chronic pain imaging studies, indicating dysfunction of pain regulation at the CNS level (51,114). It has been established that unilateral stimulation of primary motor cortex (M1) and dorsal lateral prefrontal cortex (DLPFC) with rTMS generates a diffuse analgesic effect in both experimental and clinical pain studies (115,116). The extent rTMS induced analgesic effects depend on the stimulation patterns such as the frequency and magnitudes and coil position. A single stimulation session could provide several days of analgesia, and this effect is reinforced with echoing rTMS sessions (116). This was demonstrated in Umezaki et al. with a rapid decrease in VAS scores at day 8 and 15 of rTMS treatment and a stable pain reduction score for 2 months (35). However, a peculiar finding of a temporary increase of pain score on day 30 followed by a reduction in pain score on day 60 was explained by the author as possible psycho-pathophysiological disease differences (perception of pain and duration of diseases) of each patient. Further statistical analysis shows a lack of significant improvement in the mean pain score difference for short-term rTMS used (35). rTMS is a non-invasive neuromodulation technique that could be a novel treatment in chronic pain either solely or as a complement to medication and could be useful in refractory cases. However, standardisation of therapy protocol should be established in experimental animal models before its clinical implication.

### Oral appliance (tongue protector)

A tongue protector has been shown to reduce discomfort and improve oral health and quality of life (38). BMS often presents in the anterior two-thirds of the tongue, dorsal and lateral surfaces of the tongue, anterior hard palate, lip mucosa and gingiva (4). It was thought that parafunctional habits such as tongue thrust or continuous habitual rubbing over the teeth or denture and lip, cheek, or tongue biting contribute to BMS pain (117), but this contradicts the definition of BMS (1). It is hypothesised that chronic hyperactivity of trigeminal nociceptive pathways will produce intense pain response and occurrence, or a burning mouth feeling. The use of a tongue protector may avoid other triggering factors such as dietary stimulants (hot and spicy food, citrus food) or accidental tongue irritation on the pain site. It may create a self-false psychological security belief that the appliance protects the tongue.

### Cognitive therapy

Bergdahl et al. reported an impressive reduction of three units of pain scores for both short and long-term assessment (37). The study has clearly defined its BMS patients as similar to the current ICOP recommendation (1), despite being an early years study and proven CBT benefits (37). BMS has frequently been associated with psychological disorders such as depression, anxiety, hypochondriasis and cancerphobia (4). It remains unclear whether anxiety and depression precede BMS or if they are a consequence of chronic pain. Treatment-resistant patients may have a contributing psychological factor. Cognitive behavioural therapy (CBT) is a common psychotherapeutic intervention for patients with chronic pain, and its effectiveness is influenced by the level of empathy received by the patient. Interestingly, females have commonly better outcomes than males. CBT improves the patient’s quality of life by allowing them to perform their daily activities without limitation and diverts their concentration on the pain, changing the thought and coping adaptive behaviours (118,119). A combination of psychopharmacological treatment may help the patient avoid the possibility of drug abuse and adverse effects. However, a larger sample size should be obtained to establish the benefit of CBT and to rule out the attention placebo effect, as the patient was reviewed more frequently.

In summary, the statistical analysis on the RCTs comparing intervention with placebo suggests a strong favourable outcome (SMD > 1.000) for cognitive behavioural therapy, capsaicin, topical clonazepam, and laser therapy (highest to lowest) in both short- and long-term assessment. There was some evidence on the use of phytomedicines such as umPEA, herbal catuama and hypericum perforatum in short-term pain score reduction. There were negligible changes in short term pain improvement in both trazodone and ALA (pooled effects) studies. However, the positive effects of ALA increase in long-term assessment. Although the pooled effect of ALA pain score improvement is low, the number of patients responding to ALA and its combination with gabapentin or a vitamin were high in both short- and long-term assessments. Capsaicin, topical clonazepam and saliva substitute lysozyme lactoperoxidase showed consistent treatment effectiveness or improvement in pain comparing with placebo in both short- and long-term analysis.

### Acupuncture

There is emerging interest in acupuncture as an adjunct therapy to pharmacological treatment for BMS patients due to its encouraging analgesic results on significant VAS score reduction within the first 2 months of therapy (120–125). Long-term follow-up, between 18 and 24 months after the initial acupuncture treatment, suggests a decreased level of burning sensation and improved quality of life are maintained (122,125). Scardina et al. proposed that acupuncture increases BMS patients’ lip microcirculation, which in turn reduces the localised collection of inflammatory mediators, hence providing respite from the burning pain (125). Acupuncture was not included in this review as, disappointingly, studies of this treatment to date have either been non-randomised clinical trials recruiting cohorts of consecutive BMS patients, lacked a control group, and/or administered follow up less than 2 months post-treatment. A further detailed study on the potential of acupuncture as a complementary therapy to reduce medication loading and increase patient compliance with medications is warranted.

### Limitations

There was a substantial amount of heterogeneity in the therapeutic intervention types and method of delivery. None of the included studies has a high-grade quality of evidence in both short- and long-term outcome assessment. Short-term changes in pain score, quality of life, and adverse therapy effects may not reflect the clinical practice’s real implication. Long-term outcomes data availability was minimal, with reports only on cognitive therapy, ALA, capsaicin, umPEA, topical clonazepam, and low-level laser therapy. There were other trials with similar or other treatments reported in this review but these were not included mainly due to short-term assessment of as little as 2 weeks (46,68). Publication limitation and error in the statistical study data led to limited statistical analysis comparing treatment and placebo groups. The significant efficacy of psychology and LLLT studies should be interpreted with caution due to unreported adverse effects (34,36,37). Varoni et al. is a cross-over trial assuming a sufficient wash-over period of melatonin 4 weeks before the next intervention (18). The small study samples for each group (range 10 to 33) do not provide a robust statistical power in their results. The definition of improvement or reduction in pain for categorical data analyses (RRs) were varied across the studies as some studies may have meant almost or complete recovery while other may have meant a range of numerical decrease in VAS scores.

## Conclusion

In perspective, it is suggested that multicentre trials investigate various therapeutic techniques in regulating BMS pain and increase participant numbers to conclude the treatment guidelines for BMS. The sustainability of pain reduction or remission is not adequately studied due to a short assessment period of less than a year. No treatment achieves a 50% pain remission in BMS. Investigating the influence of BMS’s biopsychosocial and neurophysiological mechanisms will provide a robust framework for integrating its various confounding aetiology factors. Studies should be ideally designed with multi-arm comparison for various pharmacological and non-pharmacological treatments to grade the treatment efficacy based on the universally accepted BMS disease’s diagnosis criteria. Likewise, a greater volume for sample size, multicentre studies, and longitudinal follow-up studies will enhance BMS treatment strategies’ value. The beneficial effects exhibited for neuroprotective and analgesic auxiliary therapies such as phytomedicine and rTMS, and the behavioural therapy CBT, could be valuable alternatives or applied in conjunction with synthetic systemic drugs, with a lesser risk of adverse drug effects, and tailored, holistic individual patient treatment, rather than the disease itself.

## Article highlights

- This paper systematically reviews the evidence base for medicines in treating BMS based on the recent ICOP definition.
- This reviews RCTs with a minimum follow-up of 2 months, which had not been conducted by any previous systematic review.
- There is evidence on the benefit of topical oral clonazepam and capsaicin and alternative medicines such as neuroprotective agents and cognitive behavioural therapy.
- There is still insufficient long term follow up on the sustainable benefits of each treatment and its side effects.
